# High prevalence of rhinitis symptoms without allergic sensitization in Estonia and Finland

**DOI:** 10.3402/ecrj.v2.25401

**Published:** 2015-04-08

**Authors:** Paula Pallasaho, Annette Kainu, Maria Juusela, Mari Meren, Anssi Sovijärvi

**Affiliations:** 1 Finnish Institute of Occupational Health, Helsinki, Finland; 2 HUCH Heart and Lung Center, Peijas Hospital, Helsinki, Finland; 3 University of Helsinki and Helsinki University Central Hospital, Vantaa, Finland; 4 Division of Clinical Physiology and Nuclear Medicine, Laboratory Department, Jorvi Hospital, Helsinki University Central Hospital, Espoo, Finland; 5 Department of Pulmonology, North Estonia Medical Centre Foundation, Tallinn, Estonia; 6 Department of Clinical Physiology and Nuclear Medicine, HUCH Medical Imaging Center, Helsinki University Central Hospital and University of Helsinki, Helsinki, Finland

**Keywords:** allergic rhinitis, allergic sensitization, chronic rhinitis, nasal polyps

## Abstract

**Objectives:**

Allergic rhinitis and atopy are more common in urban than rural environments. Non-allergic rhinitis has not been studied to a great extent. We aimed to assess the relationship of rhinitis symptoms with different profiles of allergic sensitization, comparing this in rural and urban environments.

**Methods:**

The study population consisted of population-based cohorts of adults aged 26–60 from Helsinki, Finland, and rural Saaremaa and urban Tallinn, Estonia. We compared the results of a structured interview and skin prick tests and assessed the risk factors for rhinitis.

**Results:**

The prevalence of rhinitis symptoms with atopy was 32.7% in Helsinki, 20.8% in Tallinn, and 12.5% in Saaremaa (*p*<0.001). Rhinitis symptoms without atopy were found in 26.4%, 29.8%, and 29.3% (*p*=n.s.), respectively. In Helsinki, 87.3% of participants with atopy identified symptoms as allergic, compared to 57.0% in Tallinn and 47.5% in Saaremaa. Childhood in the countryside (OR 0.63), family history of allergic rhinitis (OR 1.89), and polysensitization (OR 15.99) were significantly associated with rhinitis symptoms with atopy in a multivariate logistic regression model. The most common sensitizers were pollens and animals in Helsinki and mites in Estonia. Exposure to environmental tobacco smoke (OR 1.50) and family history of allergic rhinitis (OR 1.70) were associated with rhinitis symptoms without atopy.

**Conclusion:**

Rhinitis symptoms without allergic sensitization were common in both Finland and Estonia and were associated with environmental tobacco smoke. Family history of allergic rhinitis predisposed to rhinitis symptoms irrespective of atopy status.

Allergic rhinitis and chronic rhinosinusitis are common conditions in European populations: allergic rhinitis was reported by 26–44%, and 7–27% fulfilled the criteria for chronic rhinosinusitis in the GA2LEN survey (1). A number of studies have shown that living on a farm during childhood protects from allergic sensitization and allergic rhinitis (2–7). Other protective factors include number of siblings and contact with pets during childhood, with a dose–response pattern of protective factors and risk profiles varying by age window and atopic status (8). A report of the European Community Respiratory Health Study (ECRHS) showed that parental allergies and exposure to maternal smoking during childhood and pregnancy increases the risk for incident rhinitis (8). Smoking was associated with a high prevalence of chronic rhinitis in a recent study from Sweden (9).

In studies on the effects of rural living environment on sensitization and atopic diseases, storage mites and cockroaches were the most common sensitizers in rural villages in Mongolia (10). Studies comparing Finnish and Russian rural Karelia found no difference in sensitization to *Dermatophagoides pteronyssinus* and mugwort, while sensitization to other pollens or animals was much less common in Russia (11). Previous data from Estonia assessing differences between urban and rural environments similarly showed that cockroaches, mugwort, and storage mites mainly affected allergic rhinitis and allergic sensitization (12). However, other types of rhinitis have been studied to a much lesser extent in this respect.

We aimed to assess rhinitis symptoms in relation to different profiles of allergic sensitization, including sensitization to storage mites and cockroaches, and to compare these in rural and urban surroundings. Our hypothesis was that allergic rhinitis and atopic sensitization would be less common in Estonia than in Finland, but no difference in prevalence of rhinitis without allergic sensitization was expected between the countries irrespective of rural or urban surrounding.

## Materials and methods

### Population

The FinEsS study is a joint Nordic project between Finland, Estonia, and Sweden on respiratory epidemiology. In 1996, population-based cohorts were obtained in altogether eight locations in the three countries for a postal survey. For this study, we chose to evaluate three locations: Helsinki and Tallinn to represent urban surroundings in Finland and Estonia, and a rural environment, Saaremaa in Estonia. Of those who responded to the original postal questionnaire, subjects were randomized in each center to obtain representative samples for clinical studies. During 1997–2003, a structured interview and clinical studies including skin prick tests (SPT) with common allergens were performed. The studies were approved by the ethics committees of Helsinki University Hospital, Finland, and of Tallinn, Estonia. All participants provided signed informed consent.

### Methods

Participants underwent a structured interview on respiratory symptoms and diagnoses, smoking habits, childhood environment, and allergic conditions in the family by a physician involved in the study. SPTs were performed with 15 allergens by trained nurses according to the recommendations of the European Academy of Allergy and Clinical Immunology, but as single tests in one forearm (13). The allergens consisted of cat, dog, cow, horse, birch, timothy, mugwort, *Alternaria alternata*, *Cladosporium herbarum*, *Dermatophagoides pteronyssinus*, *Dermatophagoides farinae*, *Acarus siro*, *Lepidoglyphus destructor*, *Blattella germanica*, and latex. All extracts were provided by ALK, Hörsholm, Denmark, with the exception of latex, which was produced by Stallergenes, Antony, France. A response of at least 3 mm was regarded as positive. Each center collected their data on postal questionnaire, interview, and SPTs in data files, which were crosschecked for validity between centers, and then merged into a common database.

### Definitions

*Self-reported allergic rhinitis*: ‘Do you have or have you ever had hay fever, allergic rhinitis or allergic conjunctivitis?’

*Chronic rhinitis*: ‘Are you often bothered by a blocked or runny nose?’

*Rhinitis symptoms*: were defined as an affirmative answer to either the question on *self-reported allergic rhinitis* or *chronic rhinitis*.

*Nasal polyps*: Affirmative answer to ‘Do you have or have you had nasal polyps?’

*Atopy*: At least one positive reaction to any allergen in SPT.

*Polysensitized*: At least four positive responses to any allergens in SPT.

*Any pollen*: At least one positive response to birch, timothy or mugwort.

*Any animal*: At least one positive response to cat, dog, horse or cow.

*Any mite*: At least one positive response to Dermatophagoides pteronyssinus, Dermatophagoides farinae, Blattella germanica, Lepidoglyphus destructor or Acarus siro.

*Family history of allergic rhinitis*: Affirmative answer to ‘Have any of your parents or siblings ever had allergic eye or nose problems (hay fever)?’

*Family history of asthma*: Affirmative answer to ‘Have any of your parents or siblings ever had asthma?’

*Childhood in countryside*: Those answering ‘countryside’ to the question ‘Where did you live during the first five years of your life?’ When multiple answers were recorded, all those with affirmative answers to countryside were included.

*Furry pets*: Affirmative answer to ‘Did you have furred animals in your home or close environment before you were five years old?’

*Siblings*: ‘How many sisters or brothers do you have or have you had?’ Any number greater than zero was coded as having siblings.

*Smokers*: Those who reported current smoking or who had given up smoking less than a year ago.

*Ex-smokers*: Those having given up smoking more than a year ago.

*Eversmokers*: Smokers or ex-smokers.

*Environmental tobacco smoke (ETS)*: those who reported being currently or previously exposed to ETS either at home or at work with an affirmative answer to either of the questions ‘Are you or have you been exposed to tobacco smoke in your working environment?’ or ‘Are you or have you been exposed to tobacco smoke in your home environment?’

### Statistical analysis

All statistical analyses were conducted using the Statistical Package for Social Sciences (IBM Corp. Released 2013. IBM SPSS Statistics for Macintosh, Version 22.0. Armonk, NY: IBM Corp.). We used the Chi-square test to analyze differences between groups and Mantel-Haenzel test for trend to analyze age trends. Multiple logistic regression analyses were performed to assess the risk factors for rhinitis with and without atopy. *p* Values less than 0.05 were considered significant in all analyses.

## Results

### Study population

In Helsinki, altogether 643 of the 1,200 invited participated, and 498 SPT tests were performed. In Tallinn and Saaremaa, respectively, 1,332 and 672 were invited, of whom 579 and 450 participated. A total of 517 and 444 SPTs were performed. The age groups of the original cohorts differed according to location, but the age of 26–60 was covered overall. Participants falling outside this age range, those with missing SPTs, and those who either failed to react to the positive control (*n*=4) or reacted to the negative control (*n*=30) in SPTs, were excluded from this comparison. The characteristics of the final study samples are outlined in Table 1.

**Table 1:** Characteristics of study population

	Helsinki	Tallinn	Saaremaa	
	*n* (%)	*n* (%)	*n* (%)	*p*
Study subjects	459	379	321	
Women	261 (56.9)	203 (53.6)	177 (55.1)	0.631
Age, years, mean	44.2	44.1	43.5	
Smoking habits				
Non	199 (43.3)	164 (43.3)	162 (50.5)	0.091
Ex	105 (22.9)	73 (19.3)	49 (15.3)	0.030
Current	155 (33.8)	142 (37.5)	110 (34.3)	0.500
Family history of allergic rhinitis	206 (44.9)	81 (21.4)	62 (19.3)	<0.001
Living<5 years of age				
Town	218 (47.5)	160 (42.2)	70 (21.8)	<0.001
Suburban	92 (20.0)	82 (21.6)	19 (5.9)	<0.001
Countryside	138 (30.1)	137 (36.1)	232 (72.3)	<0.001
Mixed	11 (2.4)	0	0	n.a.
Furred pets in childhood	238 (51.9)	180 (47.5)	226 (70.4)	<0.001
Siblings	413 (90.0)	309 (81.5)	287 (89.4)	<0.001

Difference (*p* value) by area.

### Prevalence of self-reported allergic rhinitis and chronic rhinitis

The prevalence of self-reported allergic rhinitis was highest in Helsinki, at 42.3%, followed by that in Tallinn, at 24.5%, and Saaremaa, 17.1% (*p*<0.001) (Table 2). We found no difference between chronic rhinitis in the different locations: chronic rhinitis was reported by 35.7% in Helsinki, 41.7% in Tallinn, and 36.1% in Saaremaa (Table 2). Nasal polyps were most common in Tallinn, at 9.8%, with a prevalence of 5.6% in Saaremaa, and 5.4% in Helsinki (*p*=0.028). Chronic rhinitis was more common than self-reported allergic rhinitis in Estonia, whereas the opposite was true in Helsinki.

**Table 2:** Allergic sensitization profiles among all participants, and among those with self-reported allergic rhinitis and chronic rhinitis

	Helsinki	Tallinn	Saaremaa	
	*n* (%)	*n* (%)	*n* (%)	*p*
All participants				
Atopy	213 (46.4)	128 (34.0)	81 (25.2)	<0.001
Polysensitized	66 (14.4)	34 (9.0)	17 (5.3)	<0.001
Any pollen	147 (32.0)	59 (15.6)	15 (4.7)	<0.001
Any animal	130 (28.3)	44 (11.6)	19 (5.9)	<0.001
Any mite	83 (18.1)	86 (22.7)	64 (19.9)	0.253
Self-reported allergic rhinitis	194 (42.3)	93 (24.5)	55 (17.1)	<0.001
Atopy	131 (67.5)	45 (48.4)	19 (34.5)	<0.001
Polysensitized	53 (27.3)	15 (16.1)	5 (9.1)	0.005
Any pollen	104 (53.6)	26 (28.0)	4 (7.3)	<0.001
Any animal	95 (49.0)	23 (24.7)	4 (7.3)	<0.001
Any mite	41 (21.1)	29 (31.2)	18 (32.7)	0.082
Chronic rhinitis	164 (35.7)	158 (41.7)	116 (36.1)	0.161
Atopy	73 (44.5)	64 (40.5)	34 (29.3)	0.033
Polysensitized	22 (13.4)	22 (13.9)	8 (6.9)	0.153
Any pollen	52 (31.7)	30 (19.0)	4 (3.4)	<0.001
Any animal	46 (28.0)	28 (17.7)	6 (5.2)	<0.001
Any mite	21 (12.8)	41 (25.9)	31 (26.7)	0.004

Difference by location (*p* value).

### Allergic sensitization profiles

Atopy was most common in Helsinki, at 46.4%; next was that in Tallinn, at 34.0%, and Saaremaa, 25.2% (*p*<0.001) (Table 2). Among all participants, sensitization to pollens, animals, and polysensitization followed the same pattern of being most common in Helsinki and least common in Saaremaa, while no significant difference was seen between the locations regarding sensitization to any mite. Few participants were sensitized to pollens or animals in Saaremaa, but those testing positive to any mite were frequently sensitized to multiple mites. The relationship of sensitization profiles with self-reported allergic rhinitis and chronic rhinitis is demonstrated in Table 2. In Helsinki, 87.3% of participants with atopy identified symptoms as allergic, compared to 57.0% in Tallinn and 47.5% in Saaremaa.

### Rhinitis and atopy status

**Fig. 1:**
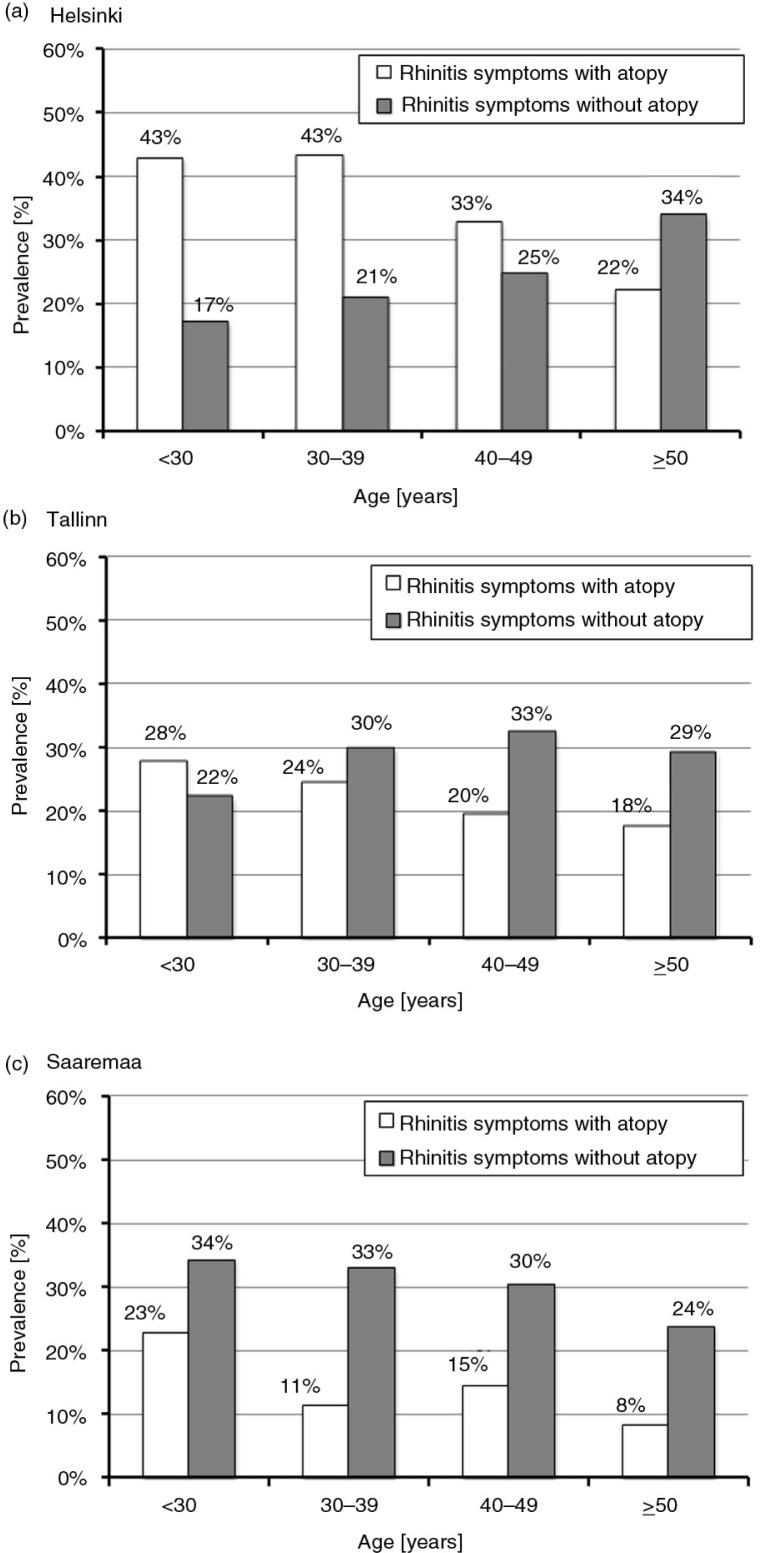
Prevalence of rhinitis symptoms with and without atopy by age decade in general population of a) Helsinki, b) Tallinn, and c) Saaremaa.

The prevalence of rhinitis symptoms by age and atopy status is shown in Fig. 1. Rhinitis symptoms without atopy were found in 26.4% in Helsinki, 29.8% in Tallinn, and 29.3% in Saaremaa (*p*=n.s.). The prevalence of rhinitis symptoms with atopy decreased with age in Helsinki (*p*<0.001), but in contrast, symptoms without atopy increased steadily with age (*p*=0.003) (Fig. 1). The decreasing trend of rhinitis symptoms with atopy in relation to age was less prominent in Tallinn. In both Estonian locations, the prevalence of symptoms without atopy seemed to be fairly stable in relation to age (*p*=n.s.).

Rhinitis symptoms with atopy were found in 32.7% in Helsinki, 20.8% in Tallinn, and 12.5% in Saaremaa (*p*<0.001). In this group, the most common sensitizer was pollen in Helsinki, at 76.0%, whereas only 15.0% of participants in Saaremaa were sensitized to pollen. Conversely, sensitization to mites (including cockroaches) was predominant in Saaremaa, with 92.5% of participants testing positive, whereas only 34.0% in Helsinki and 63.3% in Tallinn were sensitized to mites. Polysensitization was more prevalent in Helsinki at 38.0% vs. 31.6% in Tallinn and 25.0% in Saaremaa, but the difference was not statistically significant.

### Risk factors for rhinitis

Table 3 evaluates the different risk factors for rhinitis symptoms (with or without atopy) in the general population in the multivariate model stratified by location. In all locations, family history of allergic rhinitis was significantly associated with rhinitis symptoms. In addition, in Helsinki, we found significant associations for allergic sensitization to animals, pollens, and exposure to ETS. In Tallinn, the role of animals as sensitizers was strongest, with an OR of 3.18 (95% CI 1.36–7.44; *p*=0.008). In Saaremaa, the protective effect of living in the countryside in childhood was significant, with an OR of 0.58 (95% CI 0.34–0.98; *p*=0.043), in addition to a positive association with sensitization to any mite, OR 2.39 (95% CI 1.22–4.68; *p*=0.011).

**Table 3:** Multivariate logistic regression model of risk factors for rhinitis symptoms

			Helsinki			Tallinn			Saaremaa		
				
		OR	95% CI	*p*	OR	95% CI	*p*	OR	95% CI	*p*
Gender	Female	0.988	0.648–1.507	0.955	0.815	0.516–1.287	0.380	1.023	0.593–1.765	0.936
Age	Over 40 years	0.962	0.609–1.521	0.870	1.109	0.677–1.816	0.681	0.803	0.493–1.306	0.376
Childhood	In the countryside	1.061	0.634–1.777	0.821	0.619	0.381–1.005	0.052	**0.579**	**0.341–0.984**	**0.043**
	Furred pets	1.090	0.684–1.737	0.716	0.865	0.545–1.375	0.541	0.707	0.418–1.196	0.197
	Siblings	0.893	0.448–1.783	0.893	0.938	0.540–1.632	0.822	1.263	0.582–2.741	0.554
Family history	Allergic rhinitis	**2.097**	**1.351–3.254**	**0.001**	**2.260**	**1.203–4.245**	**0.011**	**2.233**	**1.090–4.576**	**0.028**
Smoking	Eversmoker	0.786	0.516–1.197	0.263	0.764	0.486–1.201	0.244	1.061	0.615–1.831	0.832
	ETS	**1.645**	**1.019–2.656**	**0.041**	1.398	0.831–2.351	0.207	1.248	0.760–2.049	0.382
Atopy	Polysensitization	2.535	0.966–6.648	0.059	1.618	0.531–4.927	0.397	1.893	0.436–8.212	0.394
	Any pollen	**1.782**	**1.011–3.142**	**0.046**	1.451	0.720–2.927	0.298	0.512	0.123–2.137	0.358
	Any animal	**2.205**	**1.197–4.062**	**0.011**	**3.176**	**1.355–7.444**	**0.008**	0.855	0.308–2.372	0.764
	Any mite	0.590	0.319–1.090	0.092	0.953	0.517–1.756	0.877	**2.390**	**1.221–4.681**	**0.011**
	Any mold	1.652	0.413–6.612	0.478	0.262	0.047–1.453	0.125	0.334	0.052–2.126	0.245

All listed variables are included in the model.

Statistically significant associations are presented in bold figures.

In the multivariate analyses shown in Table 4, rhinitis symptoms without atopy were significantly associated with exposure to ETS, OR 1.50 (95% CI 1.10–2.04; *p*=0.010), and family history of allergic rhinitis, OR 1.70 (95% CI 1.24–2.34; *p*=0.001). Rhinitis symptoms with atopy were significantly associated with a family history of allergic rhinitis, OR 1.63 (95% CI 1.17–2.27; *p*=0.004), while spending childhood in the countryside resulted in a protective effect, OR 0.62 (95% CI 0.46–0.84; *p*=0.002). When added to this model of risk factors for rhinitis with atopy, polysensitization resulted in an OR of 15.99 (95% CI 9.87–25.91; *p*<0.001).

**Table 4:** Multivariate logistic regression model of risk factors for rhinitis symptoms by atopy

	Rhinitis symptoms without atopy			Rhinitis symptoms with atopy		
		
	OR	95% CI	*p*	OR	95% CI	*p*
Location						
Helsinki	1			1		
Tallinn	1.296	0.942–1.784	0.111	**0.630**	**0.451–0.879**	**0.007**
Saaremaa	**1.518**	**1.063–2.168**	**0.022**	**0.363**	**0.239–0.552**	**<0.001**
Gender (female)	1.110	0.844–1.459	0.456	0.800	0.595–1.076	0.140
Age over 40	1.249	0.941–1.658	0.124	**0.621**	**0.460–0.838**	**0.002**
Childhood in countryside	0.892	0.658–1.208	0.459	**0.694**	**0.494–0.975**	**0.035**
Furred pets in childhood	0.810	0.610–1.077	0.148	1.189	0.871–1.625	0.276
Siblings	1.087	0.735–1.610	0.676	0.856	0.565–1.298	0.464
Eversmoker	1.040	0.791–1.367	0.781	0.795	0.592–1.069	0.129
ETS	**1.498**	**1.102–2.036**	**0.010**	0.979	0.708–1.353	0.898
Family history of allergic rhinitis	**1.700**	**1.235–2.341**	**0.001**	**1.628**	**1.168–2.268**	**0.004**
Family history of asthma	0.768	0.508–1.161	0.211	1.251	0.826–1.892	0.290

All listed variables were included in the model.

Statistically significant associations are presented in bold figures.

## Discussion

Self-reported allergic rhinitis and allergic sensitization in SPTs were more common in Helsinki, Finland, than in the Estonian locations. However, we saw no difference between chronic rhinitis in the different countries. Chronic rhinitis was reported more frequently than allergic rhinitis in Estonia; in Saaremaa the prevalence of rhinitis symptoms without atopy was over twice the prevalence of symptoms with atopy. Furthermore, nasal polyps were more common in Saaremaa. The most common sensitizers were pollens and animals in Helsinki, and mites (including cockroaches) in Estonia. Sensitization to mites was significantly associated with rhinitis symptoms in Saaremaa, Estonia, where 92.5% of those with atopy and rhinitis symptoms were sensitized to mites or cockroaches, but only 47.5% of atopic participants identified their symptoms as allergic. ETS was associated with rhinitis symptoms without atopy.

### Prevalence of allergic rhinitis, chronic rhinitis, and nasal polyps

The prevalence of self-reported allergic rhinitis in our study was consistent with previous ECRHS results that reported a prevalence of 39.9% in Finland and 17.8% in Tartu, Estonia (1, 14). Similarly, allergic rhinitis was reported by 41.6% in a population-based study from Southern Finland, with nasal polyps reported by 4.4% (15). In this study, we have shown that only 67.5% of participants with self-reported allergic rhinitis in Helsinki, 48.4% in Tallinn, and 34.5% in Saaremaa, had positive responses in SPTs, confirming atopy. Some participants may have local IgE production in the nasal epithelium and allergic rhinitis despite negative SPT results, but in a recent Spanish study the majority of such patients were non-smoking women aged <30 years (16). On the other hand, different types of rhinitis and nasal symptoms may overlap, which makes definite classification of the conditions challenging (17). Chronic rhinitis and rhinosinusitis represent multifactorial conditions with many possible underlying causes including anatomic, hormonal, environmental, infectious, and endogenous factors (18). Chronic rhinosinusitis with nasal polyps is often associated with type 2 inflammation, but evidence on neutrophilic inflammation and genetic differences regarding the inflammatory type is emerging (19). Thus, higher prevalence of nasal polyps in Estonia despite lower prevalence in allergic sensitization may be explained by different inflammatory mechanisms. The diagnostic labeling may also differ among cultures, making symptom-based comparisons unreliable. Data on different rhinitis phenotypes are scarce in the literature, and phenotyping was named one of the unmet needs in a recent ARIA (Allergic Rhinitis and its Impact on Asthma) document (20).

### Allergic sensitization

The prevalence of allergic sensitization in Helsinki, as well as in the Estonian locations, was in line with earlier results on sensitization, with a diverging sensitization profile (15, 21, 22). Sensitization to storage mites was surprisingly high in Saaremaa, but similar results have been found in other rural environments (10). Sensitization to house dust mites or cockroaches was also common among the general population in the US, and a high prevalence of storage mite sensitization has been reported in the Spanish general population (23, 24). The growth of mites has been related to temperature and humidity, as well as to older housing types (25, 26). Thus, different environments predispose to a variety of allergens, giving rise to different sensitizations. Protective factors and genetic predispositions also differ, with the effects of epigenetic mechanisms, resulting in challenging combinations of protective and sensitizing factors.

### Risk factors

Having a family history of allergic rhinitis was significantly associated with rhinitis symptoms in all locations. This may also be affected by chronic symptoms being perceived as allergic without verification of allergies. Our primary aim was to assess the association of different sensitization profiles with rhinitis types, rather than to assess childhood risk factors. A trend towards a significant negative association regarding living in the countryside during early childhood and verified allergic rhinitis was found in Tallinn and Saaremaa. When assessing the same association for rhinitis symptoms, this became significant. This negative association is consistent with the biodiversity hypothesis (27). Exposure to ETS was significantly associated with rhinitis without atopy. A higher smoking prevalence in Estonia than in Finland may contribute to the high prevalence of non-allergic rhinitis in Estonia (28).

### Strengths and limitations

Self-reported allergic rhinitis may have been underdiagnosed in Estonian participants and over diagnosed in Finnish participants since the classification was based on the answers given by the study participants during the interview. No physician diagnosis of rhinitis was obtained. The answers probably reflect the diagnostic approaches and practices of the countries. It is recommended that allergic rhinitis is diagnosed on the basis of typical symptoms and the demonstration of an IgE-mediated allergy through SPTs or specific measurements from the serum (29). When common SPT panels do not include storage mites, symptoms of rhinitis related to storage mites cannot be labeled allergic. In clinical practice, the use of allergy tests may also vary between countries. However, in this study, the cultural and diagnostic differences were compensated by evaluating participants that were found to have atopy and any reported chronic nasal symptoms. However, we recognize that atopy found in SPT does not necessarily mean that the symptoms of rhinitis are always allergic. The strengths of our study include the use of population-based random cohorts of adults representing the general population, and the use of the same methods in all locations. Another strength is the use of a broad variety of allergens in the SPTs, including storage mites and cockroaches in addition to common pollens and animals, which are not included in all comparative studies.

## Conclusion

In contrast to Finland, the prevalence of rhinitis symptoms with atopy was low in Estonia, especially in rural Saaremaa, where symptoms without atopy were twice as prevalent. Sensitization to mites or cockroaches was significantly associated with rhinitis in Saaremaa, whereas pollen or animal sensitization was not. Storage mites and cockroaches seem to be relevant allergens in Estonia, with up to 92.5% of participants with rhinitis symptoms and atopy being sensitized to mites. Thus allergy should be verified by SPTs including these allergens. Some cultural differences regarding awareness of allergic symptoms were found, their recognition being highest in Finland probably due to the national Allergy Programme. Rhinitis symptoms without sensitization were associated with exposure to ETS and with having a family history of allergic rhinitis. Phenotyping of rhinitis is needed to better understand its causes and to target treatments.
